# Epidemiological investigation and whole-genome genetic characterization of duck circovirus in Shandong Province of eastern China

**DOI:** 10.3389/fvets.2026.1949592

**Published:** 2026-09-22

**Authors:** Yuxia Zhang, Kexiang Yu, Wenbin Wang, Yuliang Xu, Kai Meng, Ye Tian, Xiaoyuan Yuan, Guiming Li

**Affiliations:** 1 Poultry Institute, Shandong Academy of Agricultural Sciences, Jinan, China; 2 Shandong Provincial Key Laboratory of Livestock and Poultry Breeding, Jinan, China

**Keywords:** *Cap* gene, duck circovirus, epidemiology, genetic analysis, *Rep* gene

## Abstract

**Introduction:**

Duck circovirus (DuCV) is a contagious immunosuppressive pathogen in duck populations, causing severe economic losses in the global waterfowl breeding industry. Shandong Province is a major duck-producing region in China, with one of the country’s largest duck populations.

**Methods:**

In this study, we systematically investigated the prevalence, genetic characteristics, and major proteins of DuCV strains found in Shandong Province. A total of 630 cloacal swab samples were collected from more than 60 duck farms across 17 cities of Shandong province in 2023. Duck circovirus and other common duck pathogens were detected by the polymerase chain reaction (PCR) method. Whole-genome sequencing was further performed to acquire three representative complete DuCV genome sequences, followed by comprehensive phylogenetic and genotypic analyses.

**Results:**

The positive rate for duck circovirus was 12.54% (79/630), with variation in detection rates among the sampled cities. *Riemerella anatipestifer* and duck parvovirus were identified as the dominant co-infecting pathogens. Genomic analysis showed that the full-genome lengths of the three complete DuCV genome sequences ranged from 1988 bp to 1993 bp. The three sequenced DuCV genome sequences belonged to the DuCV-1b and DuCV-2c sub-genotypes. Notable differences were observed in the N-glycosylation site and 3D structure of the cap proteins between the two genotypes. The three novel DuCV genome sequences shared 56.40–99.79% nucleotide sequence identity with global reference DuCV sequences. Phylogenetic trees of DuCV based on the whole genome, *Cap* gene, and *Rep* gene were constructed using reference strains from around the world.

**Conclusion:**

This study provides updated epidemiological survey data and genetic information on DuCV in Shandong Province, China, which provides reference data for further research on the prevention and control of DuCV infection.

## 1 Introduction

Duck circovirus (DuCV), a member of the genus Circovirus within the family Circoviridae, is a small, non-enveloped, single-stranded DNA virus first identified in Germany in 2003 (1). Since its initial discovery, DuCV has emerged as a significant pathogen affecting duck populations worldwide and has been reported in the United States, Korea, Poland, Hungary, and China (2–4). DuCV-infected ducks mainly exhibit symptoms such as slow growth, emaciation, anemia, and immunosuppression (5). They are prone to secondary infections by other pathogens, such as *Riemerella anatipestifer*, *E. coli*, duck hepatitis virus, and parvovirus, which cause significant economic losses in the duck-raising industry (6–9).

The genome of DuCV is approximately 1.8–2.0 kb and contains three major open reading frames (ORFs) and an untranslated region (UTR) (10). ORF1 encodes a replication-associated protein (Rep), which is involved in the replication and transcription regulation of the viral genome. ORF2 encodes the capsid protein (Cap), which forms the outer shell of the virus particle and regulates host immune responses (11), and ORF3 encodes an active apoptotic protein (12). Phylogenetic analyses based on the complete genome or the *Cap* gene have traditionally classified DuCV into three distinct genotypes, namely DuCV-1, DuCV-2, and DuCV-3. These genotypes exhibit differential geographical distribution and pathogenic potential (13, 14). DuCV-1 and DuCV-2 are further subdivided into multiple subtypes, specifically DuCV-1a to DuCV-1d and DuCV-2a to DuCV-2c. In contrast, DuCV-3 represents a novel virus identified in China in 2022 (13).

DuCV can infect multiple duck species, such as Cherry Valley (15), Muscovy (16), Pekin (17), and Muscovy duck (18), as well as wild ducks (7). Although ducks of all ages are susceptible, the highest infection rate is observed in birds aged 3 to 5 weeks (19). The infection leads to multi-organ involvement, mainly resulting in atrophy of the bursa of Fabricius and splenic lesions (20). Recent studies have indicated that co-infection with the novel goose parvovirus exerts a synergistic pathogenic effect, resulting in growth retardation and feathering disorders (21). The virus can spread vertically or horizontally and, once a duck flock is infected, it can persist within the population, causing serious economic losses in waterfowl farming. Nevertheless, large-scale molecular epidemiological surveys assessing the virus’s impact on duck farms remain relatively scarce, particularly in Shandong province, which is China’s primary duck-farming region.

Therefore, an epidemiological investigation of DuCV was conducted using samples collected from farms in Shandong province in 2023. The genetic characteristics and homology of the obtained genome sequences were analyzed against reference sequences. These findings help provide an epidemiological foundation for disease management practices, while concurrently broadening our perspective on DuCV evolution within avian populations.

## 2 Materials and methods

### 2.1 Sample collection

In 2023, a total of 60 duck farms of varying sizes across Shandong Province, China, were included in this study. Cloacal swabs were randomly collected from each farm at a sampling rate of one sample per 1,000 ducks according to flock size, yielding 630 specimens in total that covered both meat-type and breeding ducks aged 7 to 400 days. All cloacal swabs were subjected to molecular detection of DuCV. Cloacal swabs were selected as the sampling matrix because they allow non-invasive sample collection with convenient on-farm operation, and they have been widely adopted for DuCV epidemiological surveillance in duck flocks. The specific information on the samples is summarized in Supplementary Table S1.

All swab samples were immersed in sterile PBS, thoroughly vortexed, and subsequently centrifuged at 8000 × g for 10 min at 4 °C. The clarified supernatant of each sample was harvested and reserved for subsequent nucleic acid extraction.

### 2.2 Nucleic acid extraction and sample testing

The nucleic acid of the virus was extracted from the supernatant of each sample using a viral RNA/DNA extraction kit (Takara) following the manufacturer’s instructions. All samples were tested for DuCV by PCR; when a sample was found to be positive, other pathogens were further detected via PCR or RT-PCR. The primer sequences for DuCV, duck parvovirus (DPV), duck reovirus (ReoV), adenovirus type 4 (AdV), duck Tembusu virus (DTMUV), and *Riemerella anatipestifer* (RA) are shown in Table 1.

**Table 1:** Primers used for the detection of duck circovirus and other pathogens.

Primer names	Primer sequence (5′ to 3′)	Annealing temperature (°C)	Size (bp)	References
DuCV-F	CATGCCCATGCCGTAAT	55	604	(7)
DuCV-R	TCAGAAGACGAAGGCTACG			
DPV-F	CTACAACCCGGACCTGTGTC	60	922	(31)
DPV-R	GCATGCGCGTGGTCAACCTAACA			
Reov-F	GCTTTTTGAGTCCTCAGCGTG	61	1,102	(32)
Reov-R	GATGAATAGGCGAGTCCCGC			
DTMUV-F	GAAGCGAGCACCTACCACA	60	249	(33)
DTMUV-R	CGCTGATGACCCTGTCCAT			
AdV-F	CAA RTT CAG RCA GAC GGT	56	506	(34)
AdV-R	TAG TGA TGM CGS GAC ATC AT			
RA-F	TTAGATAGTTGGTGAGGTAA	58	475	(35)
RA-R	ATCGGTGTTCTGAGTAAT			

The PCR reaction system for DuCV was 25 μL, including 2 × Taq PCR Mix (Takara) 12.5 μL, F/R primers (10 μmol/L) each 1 μL, template DNA 2 μL, and ddH_2_O 8.5 μL. The PCR reaction conditions were as follows: 94 °C for 5 min; 35 cycles of 94 °C for 30 s, 55 °C for 30 s, 72 °C for 30 s; and extension at 72 °C for 5 min. The PCR amplification products were analyzed using 1% agarose gel electrophoresis and observed under ultraviolet light.

### 2.3 Whole-genome sequencing

Viral genomic DNA was extracted from some representative PCR-positive samples using a commercial viral DNA extraction kit (Tiangen). Libraries were constructed using the MGIEasy FS DNA Library Prep Kit and were sequenced on the DNBSEQ-T7 platform (BGI Genomics) with paired-end 150 bp reads and an average sequencing depth of ≥200×. Clean reads were assembled by MEGAHIT (v1.1.2) using a reference-guided strategy. Genome-terminal regions were confirmed by mapping reads across circular-genome junctions. The assembled genome sequences were further validated by mapping clean reads back to the assembled contigs. Three complete DuCV genome sequences were finally obtained. Sequencing and bioinformatic analyses were performed by BGI Genomics Co., Ltd.

### 2.4 Genetic sequence analysis

The obtained whole-genome sequences and core genes, such as *Rep* and *Cap*, were subjected to homology comparisons and evolutionary analyses with sequences from different countries. The sequences of all reference strains used were obtained from the National Center for Biotechnology Information (NCBI) database at https://www.ncbi.nlm.nih.gov/genbank/. The specific serial numbers are shown in Figures 1–3, respectively.

**Figure 1:**
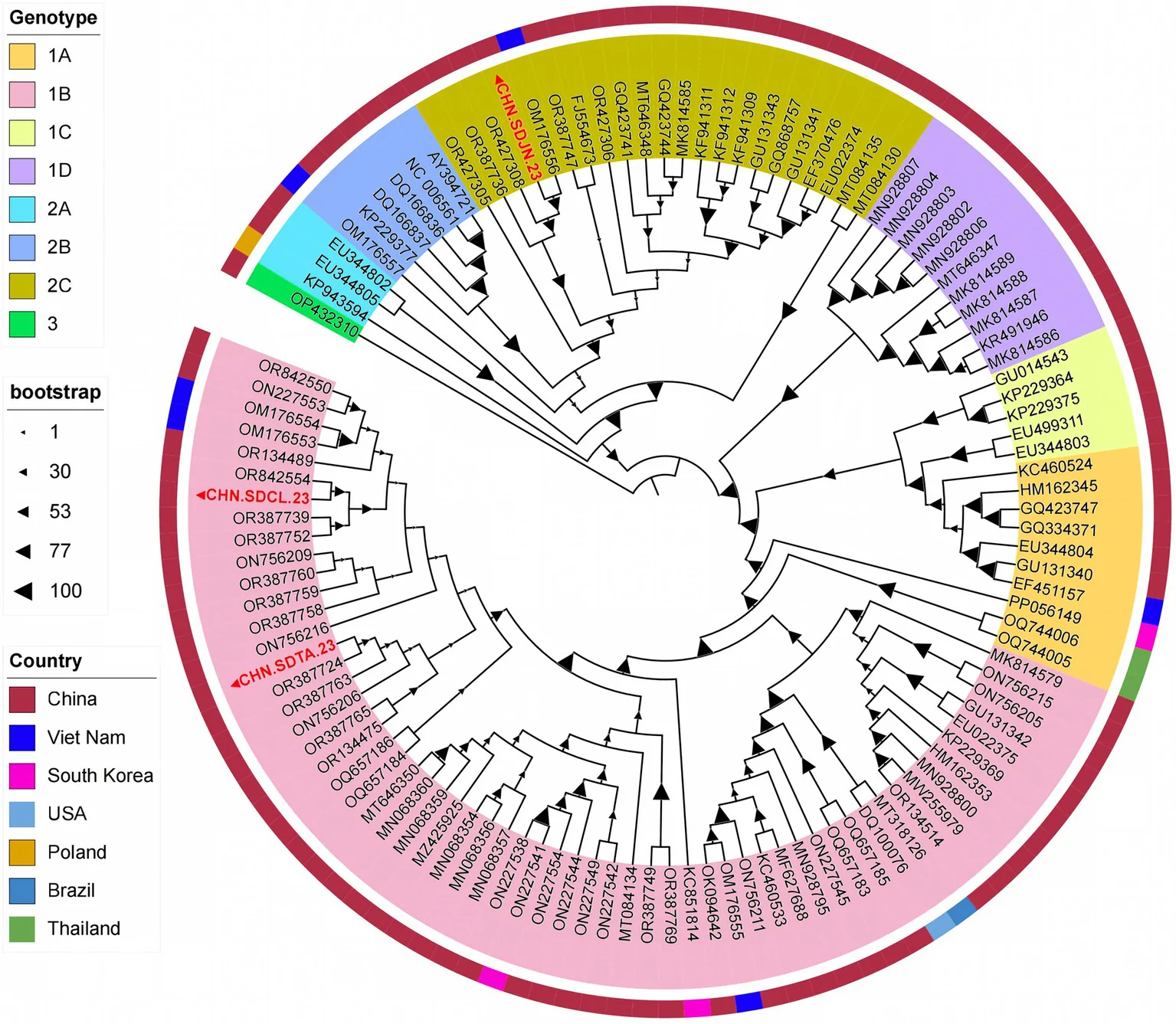
Phylogenetic tree of duck circovirus based on the whole genome.

**Figure 2:**
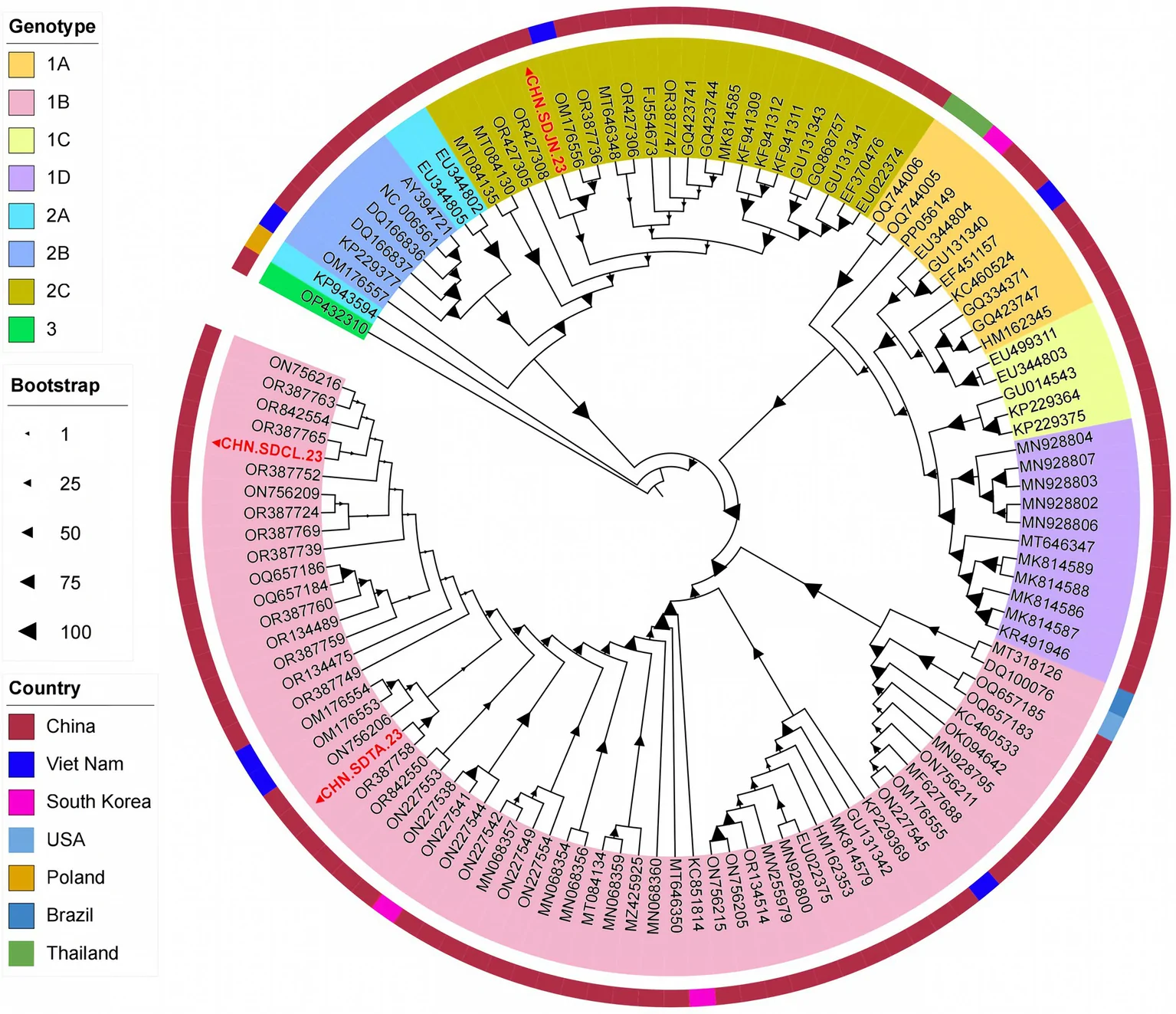
Phylogenetic tree of duck circovirus based on the Cap protein.

**Figure 3:**
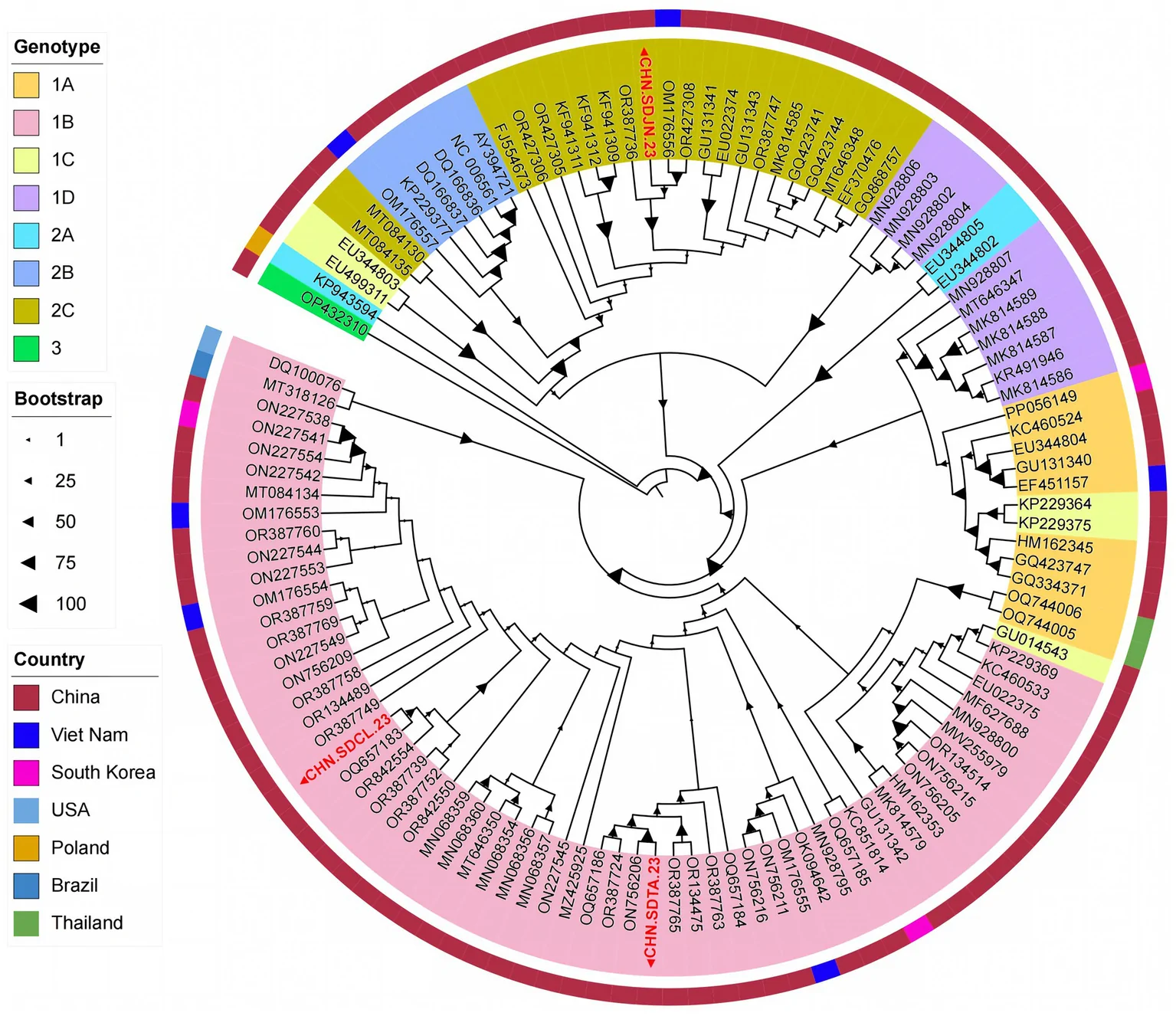
Phylogenetic tree of Duck Circovirus based on the Rep protein.

The evolutionary history was inferred using the neighbor-joining method (22). An optimal phylogenetic tree was constructed, with bootstrap values based on 1,000 replicates displayed beside the branches to represent the percentage of replicates in which the associated taxa clustered together (23). The tree is drawn to scale, with branch lengths measured in the same units as the evolutionary distances used to infer the phylogeny. These distances were calculated using the Kimura 2-parameter method (24) and are expressed as the number of base substitutions per site. All ambiguous positions were removed for each sequence pair using pairwise deletion. The evolutionary analyses were conducted using MEGA X (25).

### 2.5 Amino acid site analysis and prediction of protein structure

Multiple sequence alignment via Clustal Omega was employed to identify amino acid insertions, deletions, or substitutions responsible for structural differences between the three strains. The online software program NetNGlyc1.0^1^ was used to analyze the N-glycosylation sites of the Cap protein of DuCV strains.

The three-dimensional structures of the rep and cap proteins from the isolation strains were predicted using AlphaFold Server 3 with default settings. For each protein, five models were generated, and the one with the highest pLDDT score was selected for analysis. Structural visualization, domain architecture examination, and comparison of catalytic site exposure, terminal peptide disposition, NLS helix conformation, and antigenic loop orientation were performed using PyMOL. Recombination detection was performed using Recombination Detection Program 4.

## 3 Results

### 3.1 Prevalence of DuCV

Samples were collected from 60 duck-raising farms across 17 cities in Shandong province and tested. Of these, 22 farms were identified as DuCV-positive, resulting in a positivity rate of 36.67%. Out of 630 cloacal swabs, 79 tested positive for DuCV, with a positivity rate of 12.54%. A positive distribution map of cloacal swab samples is shown in Figure 4. In particular, Heze had the highest number of DuCV-positive cases (13), followed by Jining (12), while the samples from Weihai were negative.

**Figure 4:**
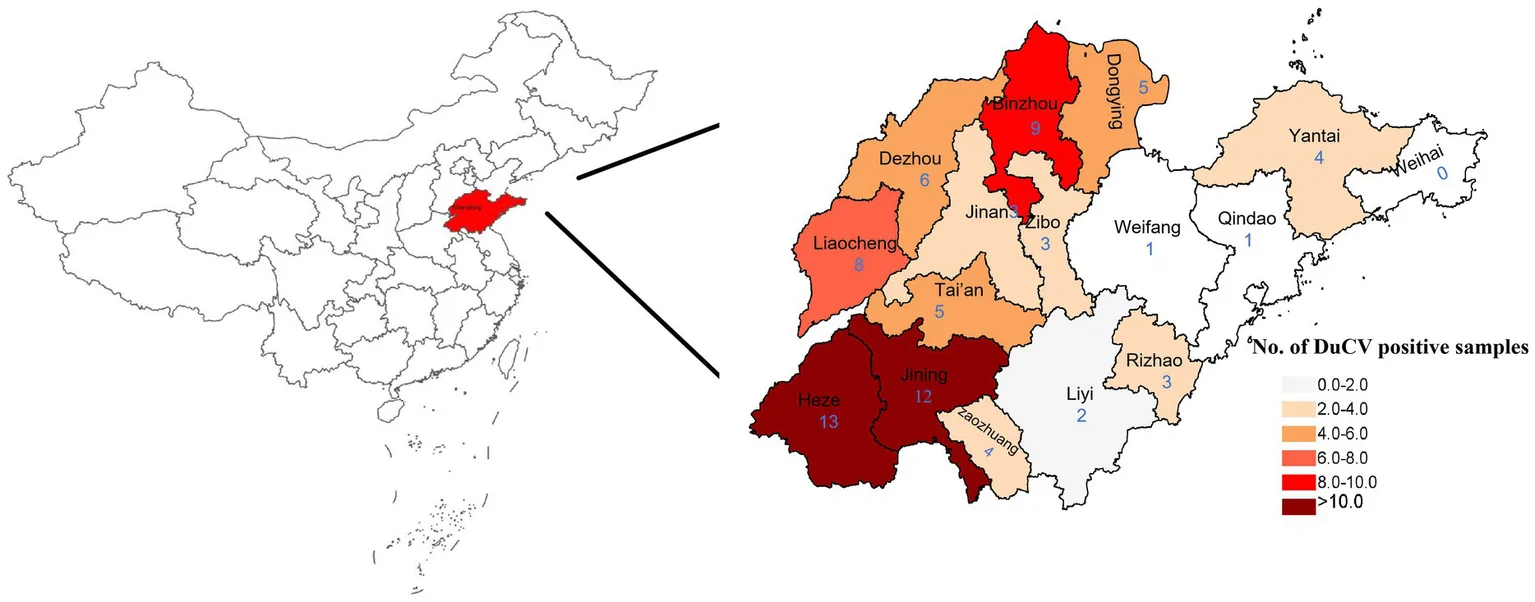
Distribution map shows the isolation locations of the positive samples.

In the present study, ducks aged 21–30 days exhibited the highest positive rate for DuCV, accounting for 38% (30/79) of all DuCV-positive samples, followed by a 24% (19/79) positivity rate among ducks aged 31–40 days. The oldest DuCV-positive duck detected was 310 days of age (Figure 5). In this study, five pathogens frequently reported to co-infect with DuCV were detected. Among the 79 samples identified as positive for DuCV, 12 were confirmed as co-infected, resulting in a co-infection rate of 15.2% (12/79). *Riemerella anatipestifer* and duck parvovirus were the predominant co-infecting agents across all tested samples, with five and four co-infection cases detected, respectively. Other pathogens, including duck DTMUV and AdV, were also detected sporadically. Notably, no reovirus infections or triple co-infections with three distinct pathogens were detected in this study.

**Figure 5:**
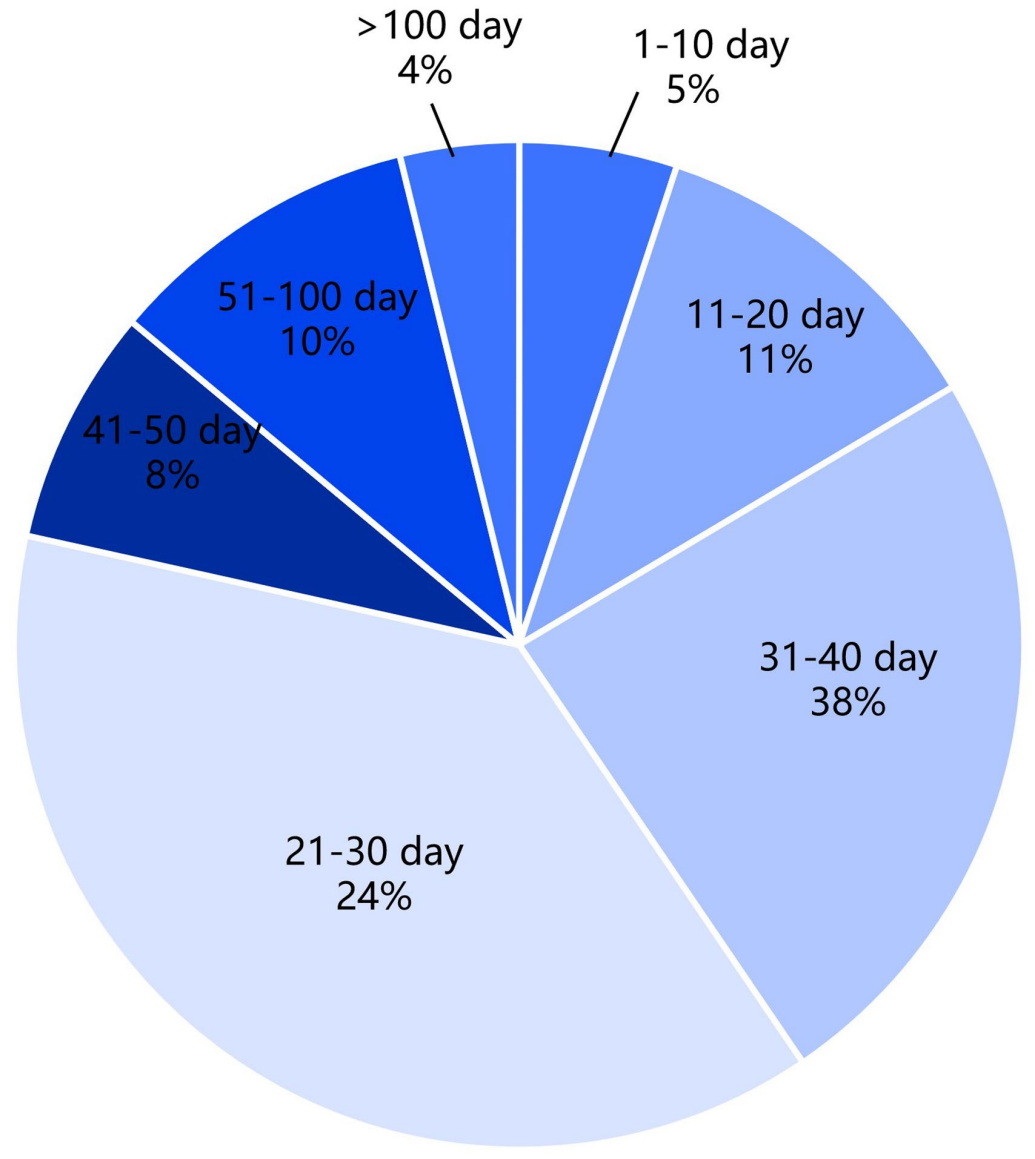
Distribution of the number of DuCV-positive samples relative to the number of day-old DuCV-infected ducks.

### 3.2 Genetic characterization

In the study, three DuCV strains were randomly selected from PCR-positive samples identified on farms in Changle (Weifang), Jinan, and Tai’an to perform complete genome sequencing. The full-length genomic sequences obtained were submitted to GenBank corresponding to CHN strains. These strains were designated as SDCL23 (abbreviated as CL), CHN. SDTA23 (abbreviated as TA), and CHN. SDJN23 (abbreviated as JN), with accession numbers PV413215–PV413217. Consistent with other DuCV, all three DuCV genome sequences contained three ORFs, with total genome lengths ranging from 1988 bp to 1993 bp.

A total of 117 globally available DuCV reference sequences from endemic countries (China, Vietnam, South Korea, the United States, Poland, Brazil, and Thailand) were included for phylogenetic and homology analysis (Figure 1). Geographically, DuCV exhibited a worldwide distribution, with the majority of sequences originating from China due to its large-scale duck breeding industry. DuCV strains were widely prevalent across East and Southeast Asian countries and sporadically detected in non-Asian regions. Sequence identity analyses of Cap, Rep, and complete genome sequences from the three DuCV sequences were conducted against 117 reference strains, and the statistical results are summarized in Table 2. The whole-genome homology analysis revealed that the three novel DuCV sequences shared 99.52–99.79% maximum nucleotide identity with global DuCV references, while the lowest identity (56.40–56.71%) was observed when these sequences were compared with DuCV3 strains. The phylogenetic analysis revealed that the JN sequence clustered within genotype 2C, whereas both the CL and TA sequences belonged to genotype 1B.

**Table 2:** Nucleotide sequence identity statistics of Cap, Rep, and complete genome sequences from three Shandong DuCV sequences versus 117 reference strains.

Region/Gene	Strain	Highest homology (%)	Minimum homology (%)	Average homology (%)	Proportion of strains with homology ≥ 95%
Cap	CHN.SDCL.23	99.87	51.73	90.75	48/117
	CHN.SDJN.23	99.74	51.56	83.70	29/117
	CHN.SDTA.23	99.35	52.08	90.53	48/117
Rep	CHN.SDCL.23	99.89	83.67	95.67	79/117
	CHN.SDJN.23	99.55	83.79	90.61	27/117
	CHN.SDTA.23	99.66	84.01	95.42	79/117
WGS	CHN.SDCL.23	99.79	56.40	91.95	65/117
	CHN.SDJN.23	99.70	56.70	85.13	28/117
	CHN.SDTA.23	99.52	56.40	91.70	61/117

Furthermore, the whole-genome homology coverage analysis revealed variable genetic conservation among the three DuCV sequences. CL possessed the highest coverage, with more than half of the reference strains showing ≥95% homology and approximately 70% showing ≥90% homology. In contrast, JN exhibited an extremely low coverage of highly homologous strains, with less than one-quarter of the reference strains showing ≥95% homology (Figure 1).

The genetic homology of the major *Cap* and *Rep* genes was further analyzed to clarify the molecular characteristics of the three DuCV sequences. For the *Cap* gene, the three strains exhibited nucleotide identities of 51.73–99.87% (CL), 51.56–99.74% (JN), and 52.08–99.35% (TA) against the 117 reference strains. CL and TA showed average *Cap* gene homology exceeding 95%, indicating high genetic conservation with prevailing field DuCV strains. Notably, all three DuCV sequences shared the lowest genetic similarity (approximately 51.5–52.1%) with the reference strain OP432310, suggesting substantial genetic divergence between the current sequences and this distant strain (Figure 2).

For the *Rep* gene, the sequence similarities of CL, JN, and TA with the reference strains ranged from 83.67–99.89%, 83.79–99.55%, and 84.01–99.66%, respectively, indicating high nucleotide homology. The phylogenetic analysis of the *Rep* gene demonstrated that CL and TA clustered in the same clade with extremely close genetic relationships, whereas JN formed an independent branch, indicating distinct evolutionary characteristics (Figure 3).

No obvious cross-genotype recombination events were detected in the *Rep* gene, *Cap* gene, or intergenic region of the three isolates. Although they belonged to different genotypes, no chimeric recombination patterns with heterogeneous origins in any gene region were observed. Nevertheless, the co-circulation of multiple DuCV genotypes increases the ecological potential for co-infection and recombination in this region.

### 3.3 Amino acid site analysis of cap protein

The amino acid residues in the Cap protein of DuCV, namely 47, 55, 82, 106, 107, 183, 194, 197, 205, and 236, are all located in the flexible antigen loop region on the surface of the capsid protein, which is a core hotspot for viral evolution and N-glycosylation regulation.

The TA and CL strains exhibited complete amino acid conservation at all 10 key sites, indicating highly consistent immunogenic profiles between the two strains. In contrast, the JN strain harbored five critical amino acid mutations, including N → H at site 47, N → S at site 55, T → G at site 194, R → K at site 205, and D → E at site 236 (Figure 6). The variation may result in the loss of one N-glycosylation site in the JN strain. Variations at the above-mentioned sites may cause the viruses to fall into two distinct evolutionary clusters.

**Figure 6:**
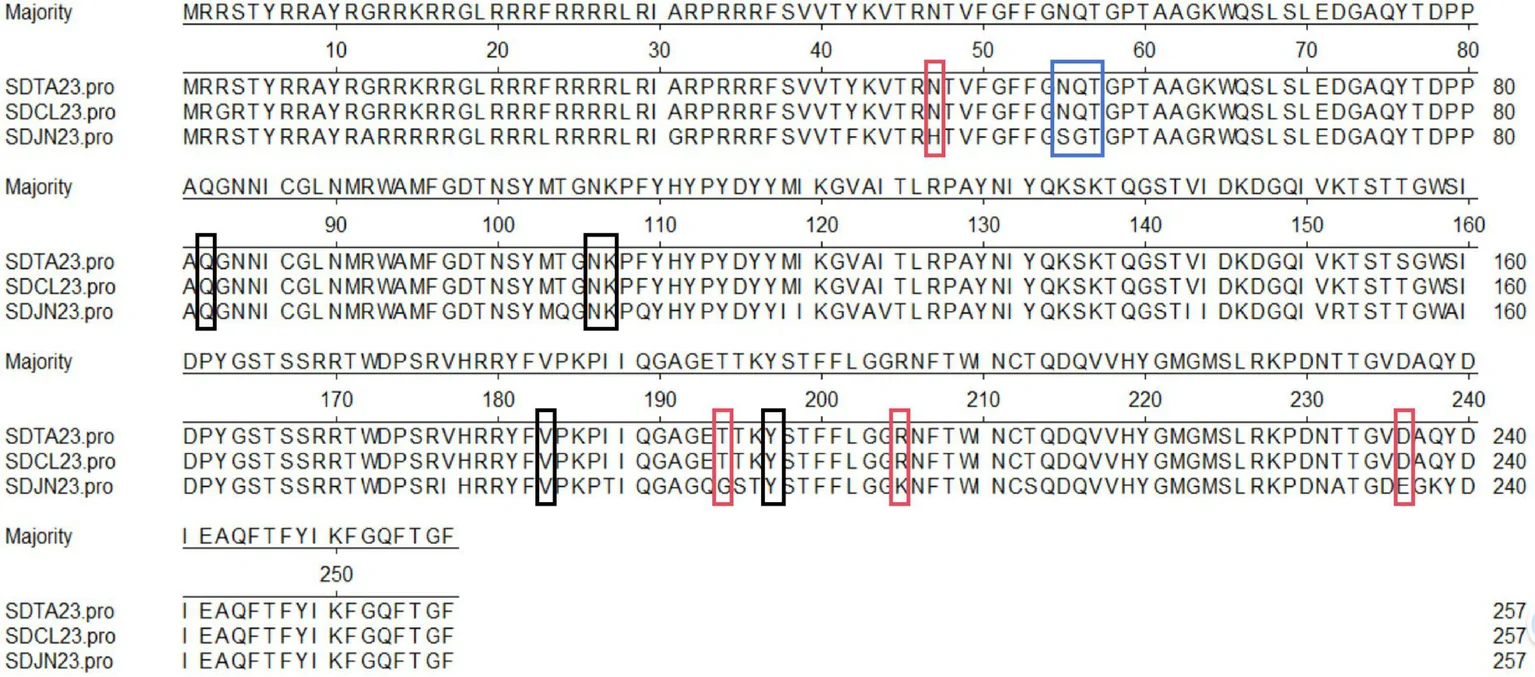
N-linked glycosylation and mutations of some amino acid sites in the Cap protein. The markers in red boxes indicate the sites with differences, while those in black boxes remain unchanged, and N-glycosylation motifs are in blue boxes.

### 3.4 Three-dimensional structure prediction of the primary protein

The structure predicted by AlphaFold reveals that the CL and TA strains have similar conformations, but differ significantly from the JN strain. For Rep proteins, CL and TA strains adopted an extended dumbbell-like shape with dispersed domains, an open endonuclease groove exposing the catalytic site, and outwardly free terminal peptides (Figure 7A). In contrast, the JN strain was compactly folded with N- and C-termini closely apposed and terminal peptides buried internally (Figure 7B). For cap proteins, both strains shared an N-terminal NLS helix and a C-terminal jelly-roll β-barrel, but CL and TA strains displayed a short, compact NLS helix with a short-disordered linker (Figure 7C). In contrast, JN exhibited a longer, more extended NLS helix and a markedly elongated linker due to amino acid insertions, deletions, or substitutions in the NLS region (Figure 7D). The C-terminal jelly-roll domain was conserved in both strains, but the antigenic loop was inwardly retracted with buried epitopes in DuCV-1. However, in DuCV-2, key neutralizing epitope-bearing loops were outwardly exposed due to residue substitutions.

**Figure 7:**
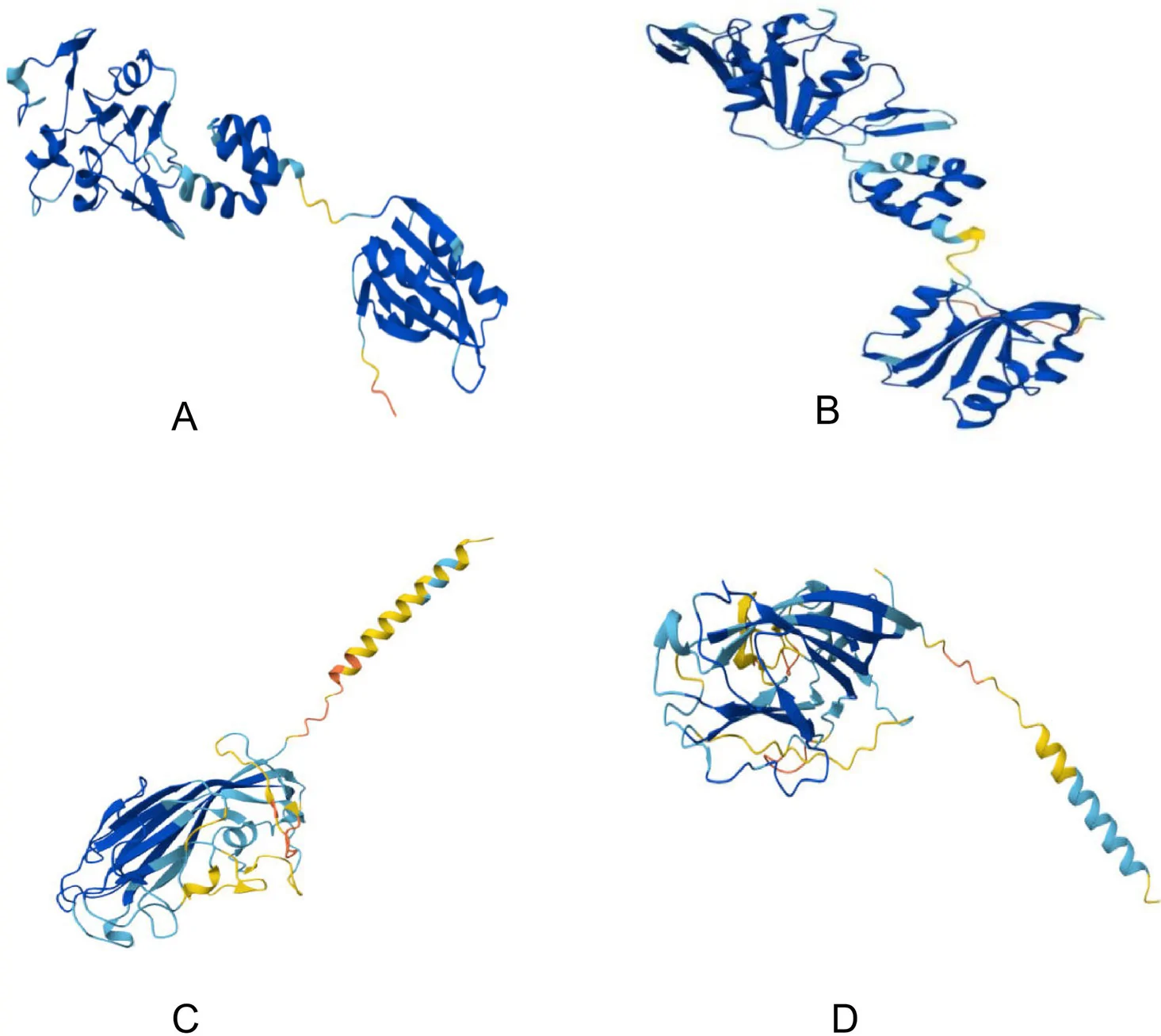
Three-dimensional structure prediction of the Rep and Cap proteins using AlphaFold 3. **(A)** Rep protein of CL strain, **(B)** Rep protein of JN strain, **(C)** Cap protein of CL strain, and **(D)** Cap protein of JN strain.

## 4 Discussion

In the present study, an epidemiological investigation of DuCV was performed using cloacal swabs collected in duck farms across Shandong Province. The positive rate of DuCV was 12.54%, with positive samples detected in 22 of 60 surveyed farms. This positive rate is relatively low compared to previous reports from other countries or provinces in China (26, 27). Notably, this study used only non-invasive cloacal swabs from live ducks rather than tissue samples from diseased or dead ducks, which typically contain higher viral loads. Given that cloacal shedding is a major DuCV excretion route, PCR-positive cloacal swabs only confirm viral nucleic acid detection. These results show that DuCV occurs in apparently healthy live ducks across multiple Shandong Province farms. Asymptomatic carrier ducks can serve as key hidden reservoirs for intra-farm horizontal transmission and are easily missed in routine disease surveillance.

Three full-length DuCV genomes were successfully sequenced from 79 cloacal swabs that tested PCR-positive. A global distribution map of the full-length genomes and the key genes of DuCV was drawn to provide a reference for the formulation of preventive and control strategies. Previous nationwide epidemiological investigation and phylogenetic analysis of DuCV in China indicated that DuCV-1b and DuCV-2c have become the dominant circulating genotypes since 2021, with DuCV-2c predominantly prevalent in southern China duck farms (7). Consistently, the phylogenetic analysis in our study assigned one isolate to Genotype 2c and the remaining two to Genotype 1b, confirming co-circulation of multiple DuCV genotypes in Shandong duck flocks. Other subtypes of the virus have not been detected, such as the 1d genotype virus found in Anhui (28), which is attributed to the limited number of samples for whole-genome sequencing in this study.

Currently, the absence of a stable *in vitro* viral culture system has hindered the development of commercial vaccines against duck circovirus. There have been studies promoting the development of DNA vaccines using cap proteins, which have shown strong immunogenicity (29). However, there are also studies showing that the majority of mutations occur at the cap protein epitope, and genomic distance genotype thresholds between different genotypes are so low they could even be considered as two types of viruses (14). The cap protein is the only structural and core immunogenic protein of DuCV. It self-assembles into viral capsids, facilitates nuclear trafficking of viral genomes, and mediates viral adhesion to host-cell receptors to complete infection. The amino acid residues in the cap protein, such as 55, 82, and 106, are located on the linear surface of B cells; changes at these sites may affect the antigenicity of the virus (7). N-glycosylation is a pivotal post-translational modification that modulates viral antigen presentation and immune recognition (30). In this study, the loss of one N-glycosylation site in the JN strain, combined with the unique amino acid substitutions in the antigenic loop region, may alter the antigenic properties of the JN strain relative to TA and CL strains.

Structural variations within the Rep protein highlight distinct potential adaptive strategies among these viral strains. The open catalytic groove of the CL strain Rep likely strengthens endonuclease activity and replication efficiency, whereas the compact folded conformation of the JN Rep protein may inhibit its enzymatic activity. Further functional investigations are required to clarify the biological consequences of this structural feature during DuCV infection. These site-specific and structural alterations may influence DuCV antigenicity or immunogenicity. However, such functional hypotheses require experimental validation. Our observations provide insights for understanding different DuCV genotypes.

## 5 Conclusion

This study provides updated epidemiological survey data and genetic information on DuCV in Shandong Province, China. The results indicate a moderate prevalence of the virus alongside a widespread subclinical infection, with the highest positive rate observed in ducks aged 20–40 days. *Riemerella anatipestifer* and duck parvovirus were identified as the predominant co-infecting agents. A global distribution map of the full-length genomes and the key genes of DuCV was created. Genetic evolutionary analysis revealed that genotypes 1b and 2c were identified in this survey conducted in Shandong province. Furthermore, a structural analysis of the Cap and Rep proteins was conducted for the two genotypes. Collectively, these findings provide important data for a deeper understanding of the epidemiological characteristics, genetic features, and global distribution of DuCV.

## Data Availability

The data analyzed in this study is available in the NCBI Genbank (https://www.ncbi.nlm.nih.gov/genbank) under accession numbers PV413215–PV413217.

## Footnotes

1
https://services.healthtech.dtu.dk/services/NetNGlyc-1.0/
