# Supplementary material for: Epidemiological investigation and whole-genome genetic characterization of duck circovirus in Shandong Province of eastern China
Source: Front Vet Sci. 2026 Sep 22;13:1949592. doi: 10.3389/fvets.2026.1949592 (PMC13639962; doi:10.3389/fvets.2026.1949592)
Supplement: Supplementary file 1 [file Table_1.docx]

**Table S1. Summary of sample collection and testing information for this study**

| Farm code | Sample collection date | Region | Breed | age | No. of samples collected | No of DuCV-postive | No of Ducv-postive | No of DPV-positive | No of ReoV-positive | No of DTMUV-positive | No of AdV-positive | No of RA-positive |
| --- | --- | --- | --- | --- | --- | --- | --- | --- | --- | --- | --- | --- |
| 1 | 2023.01 | Jining | Muscovy Duck | 15 | 10 | 1 | 12 | 0 | 0 | 1 | 0 | 0 |
| 2 | 2023.07 |  | White Duck | 30 | 10 | 8 |  | 0 | 0 | 0 | 0 | 0 |
| 3 | 2023.10 |  | Cherry Valley Duck | 28 | 5 | 3 |  | 1 | 0 | 0 | 0 | 0 |
| 4 | 2023.02 | Jinan | White Duck | 42 | 10 | 2 | 3 | 0 | 0 | 0 | 0 | 0 |
| 5 | 2023.04 |  | Cherry Valley Duck | 7 | 10 | 0 |  | 0 | 0 | 0 | 0 | 0 |
| 6 | 2023.06 |  | Muscovy Duck | 10 | 8 | 0 |  | 0 | 0 | 0 | 0 | 0 |
| 7 | 2023.09 |  | Cherry Valley Duck | 30 | 10 | 0 |  | 0 | 0 | 0 | 0 | 0 |
| 8 | 2023.11 |  | Cherry Valley Duck | 30 | 5 | 1 |  | 0 | 0 | 0 | 0 | 0 |
| 9 | 2023.02 | Zibo | / | 10 | 10 | 0 | 3 | 0 | 0 | 0 | 0 | 0 |
| 10 | 2023.06 |  | Muscovy Duck | 12 | 10 | 3 |  | 0 | 0 | 1 | 0 | 0 |
| 11 | 2023.10 |  | Cherry Valley Duck | 40 | 20 | 0 |  | 0 | 0 | 0 | 0 | 0 |
| 12 | 2023.12 |  | / | 25 | 10 | 0 |  | 0 | 0 | 0 | 0 | 0 |
| 13 | 2023.02 | Zao  zhuang | White Duck | 60 | 7 | 2 | 4 | 0 | 0 | 0 | 0 | 0 |
| 14 | 2023.07 |  | / | 120 | 10 | 0 |  | 0 | 0 | 0 | 0 | 0 |
| 15 | 2023.11 |  | White Duck | 100 | 10 | 2 |  | 0 | 0 | 0 | 0 | 0 |
| 16 | 2023.01 | Qing  dao | / | 48 | 10 | 1 | 1 | 0 | 0 | 0 | 0 | 0 |
| 17 | 2023.07 |  | / | 46 | 10 | 0 |  | 0 | 0 | 0 | 0 | 0 |
| 18 | 2023.01 | Tai'an | White Duck | 40 | 10 | 0 | 5 | 0 | 0 | 0 | 0 | 0 |
| 19 | 2023.04 |  | Cherry Valley Duck | 38 | 15 | 3 |  | 0 | 0 | 0 | 0 | 1 |
| 20 | 2023.06 |  | Muscovy Duck | 38 | 10 | 2 |  | 0 | 0 | 0 | 0 | 0 |
| 21 | 2023.09 |  | Cherry Valley Duck | 30 | 10 | 0 |  | 0 | 0 | 0 | 0 | 0 |
| 22 | 2023.11 |  | / | 30 | 10 | 0 |  | 0 | 0 | 0 | 0 | 0 |
| 23 | 2023.02 | Wei  fang | / | 280 | 15 | 1 | 1 | 0 | 0 | 0 | 0 | 0 |
| 24 | 2023.08 |  | White Duck | 400 | 10 | 0 |  | 0 | 0 | 0 | 0 | 0 |
| 25 | 2023.02 | Weihai | Cherry Valley Duck | 18 | 10 | 0 | 0 | 0 | 0 | 0 | 0 | 0 |
| 26 | 2023.08 |  | Cherry Valley Duck | 21 | 10 | 0 |  | 0 | 0 | 0 | 0 | 0 |
| 27 | 2023.11 |  | White Duck | 35 | 10 | 0 |  | 0 | 0 | 0 | 0 | 0 |
| 28 | 2023.02 | Yantai | / | 15 | 10 | 0 | 4 | 2 | 0 | 0 | 0 | 0 |
| 29 | 2023.08 |  | Muscovy Duck | 7 | 10 | 4 |  | 0 | 0 | 0 | 0 | 0 |
| 30 | 2023.12 |  | / | 18 | 10 | 0 |  | 0 | 0 | 0 | 0 | 0 |
| 31 | 2023.02 | Rizhao | / | 40 | 15 | 0 | 3 | 0 | 0 | 0 | 0 | 0 |
| 32 | 2023.06 |  | / | 30 | 10 | 3 |  | 0 | 0 | 0 | 0 | 0 |
| 33 | 2023.11 |  | / | 28 | 15 | 0 |  | 0 | 0 | 0 | 0 | 0 |
| 34 | 2023.01 | Heze | Cherry Valley Duck | 30 | 10 | 6 | 13 | 0 | 0 | 0 | 0 | 0 |
| 35 | 2023.04 |  | Cherry Valley Duck | 210 | 10 | 0 |  | 0 | 0 | 0 | 0 | 0 |
| 36 | 2023.09 |  | Muscovy Duck | 30 | 14 | 4 |  | 0 | 0 | 0 | 0 | 1 |
| 37 | 2023.12 |  | Cherry Valley Duck | 46 | 10 | 3 |  | 0 | 0 | 0 | 1 | 0 |
| 38 | 2023.02 | Dong  ying | White Duck | 40 | 10 | 3 | 5 | 1 | 0 | 0 | 0 | 0 |
| 39 | 2023.06 |  | / | 120 | 10 | 1 |  | 0 | 0 | 0 | 0 | 0 |
| 40 | 2023.11 |  | / | 310 | 15 | 1 |  | 0 | 0 | 0 | 0 | 0 |
| 41 | 2023.01 | Dezhou | Cherry Valley Duck | 40 | 10 | 0 | 6 | 0 | 0 | 0 | 0 | 0 |
| 42 | 2023.04 |  | Cherry Valley Duck | 35 | 10 | 4 |  | 0 | 0 | 0 | 0 | 2 |
| 43 | 2023.06 |  | Cherry Valley Duck | 36 | 10 | 0 |  | 0 | 0 | 0 | 0 | 0 |
| 44 | 2023.09 |  | Muscovy Duck | 35 | 13 | 0 |  | 0 | 0 | 0 | 0 | 0 |
| 45 | 2023.11 |  | Muscovy Duck | 40 | 10 | 2 |  | 0 | 0 | 0 | 0 | 0 |
| 46 | 2023.01 | Linyi | / | 28 | 10 | 0 | 2 | 0 | 0 | 0 | 0 | 0 |
| 47 | 2023.04 |  | White Duck | 30 | 10 | 0 |  | 0 | 0 | 0 | 0 | 0 |
| 48 | 2023.06 |  | / | 30 | 10 | 2 |  | 0 | 0 | 0 | 0 | 0 |
| 49 | 2023.09 |  | / | 28 | 10 | 0 |  | 0 | 0 | 0 | 0 | 0 |
| 50 | 2023.11 |  | / | 30 | 13 | 0 |  | 0 | 0 | 0 | 0 | 0 |
| 51 | 2023.12 |  | / | 9 | 10 | 0 |  | 0 | 0 | 0 | 0 | 0 |
| 52 | 2023.01 | Liao  cheng | Cherry Valley Duck | 15 | 10 | 5 | 8 | 1 | 0 | 0 | 0 | 0 |
| 53 | 2023.04 |  | Cherry Valley Duck | 18 | 15 | 0 |  | 0 | 0 | 0 | 0 | 0 |
| 54 | 2023.06 |  | / | 26 | 10 | 3 |  | 0 | 0 | 0 | 0 | 0 |
| 55 | 2023.09 |  | Muscovy Duck | 26 | 10 | 0 |  | 0 | 0 | 0 | 0 | 0 |
| 56 | 2023.11 |  | / | 30 | 10 | 0 |  | 0 | 0 | 0 | 0 | 0 |
| 57 | 2023.02 | Binzhou | Cherry Valley Duck | 42 | 5 | 0 | 9 | 0 | 0 | 0 | 0 | 0 |
| 58 | 2023.06 |  | Cherry Valley Duck | 40 | 10 | 5 |  | 0 | 0 | 0 | 0 | 1 |
| 59 | 2023.10 |  | White Duck | 100 | 10 | 4 |  | 0 | 0 | 0 | 0 | 0 |
| 60 | 2023.12 |  | / | 36 | 10 | 0 |  | 0 | 0 | 0 | 0 | 0 |
| Total |  |  |  |  | 630 | 79 | 79 | 5 | 0 | 2 | 1 | 4 |
